# Brain-Computer Interface Training With Functional Electrical Stimulation: Facilitating Changes in Interhemispheric Functional Connectivity and Motor Outcomes Post-stroke

**DOI:** 10.3389/fnins.2021.670953

**Published:** 2021-09-27

**Authors:** Anita M. Sinha, Veena A. Nair, Vivek Prabhakaran

**Affiliations:** ^1^Department of Biomedical Engineering, University of Wisconsin-Madison, Madison, WI, United States; ^2^Department of Radiology, University of Wisconsin-Madison, Madison, WI, United States

**Keywords:** brain-computer interface, stroke, upper extremity, resting-state fMRI, neurorehabilitation, motor recovery

## Abstract

While most survivors of stroke experience some spontaneous recovery and receive treatment in the subacute setting, they are often left with persistent impairments in upper limb sensorimotor function which impact autonomy in daily life. Brain-Computer Interface (BCI) technology has shown promise as a form of rehabilitation that can facilitate motor recovery after stroke, however, we have a limited understanding of the changes in functional connectivity and behavioral outcomes associated with its use. Here, we investigate the effects of EEG-based BCI intervention with functional electrical stimulation (FES) on resting-state functional connectivity (rsFC) and motor outcomes in stroke recovery. 23 patients post-stroke with upper limb motor impairment completed BCI intervention with FES. Resting-state functional magnetic resonance imaging (rs-fMRI) scans and behavioral data were collected prior to intervention, post- and 1-month post-intervention. Changes in rsFC within the motor network and behavioral measures were investigated to identify brain-behavior correlations. At the group-level, there were significant increases in interhemispheric and network rsFC in the motor network after BCI intervention, and patients significantly improved on the Action Research Arm Test (ARAT) and SIS domains. Notably, changes in interhemispheric rsFC from pre- to both post- and 1 month post-intervention correlated with behavioral improvements across several motor-related domains. These findings suggest that BCI intervention with FES can facilitate interhemispheric connectivity changes and upper limb motor recovery in patients after stroke.

## Introduction

Approximately 800,000 people experience a new or recurrent stroke in the United States each year (Benjamin et al., 2017). An estimated 80% of survivors live with upper extremity hemiparesis that significantly impacts their independence in performing daily activities and overall quality of life (Brauer et al., 2013), constituting stroke as a leading cause of acquired long-term disability. During the recovery phase of stroke, the primary standards of care include physiotherapy and/or occupational therapy. Unfortunately, these treatments only provide patients with partial recovery of motor function, resulting in learned non-use of the affected limb and eventual further loss of motor function (Ballester et al., 2016). To address and solve this unmet need for more effective therapies, there is a concerted effort to develop alternative approaches to restore upper limb motor function post-stroke. Several innovative therapeutic strategies, such as transcranial direct current stimulation (Kang et al., 2016), mirror therapy (Michielsen et al., 2011), robot-assisted training (Trujillo et al., 2017; Vahdat et al., 2018), and constraint-induced movement therapy (Lang et al., 2013) have emerged as promising techniques for stroke rehabilitation. Despite encouraging results shown by these and other studies, there is large variability in reported changes of neuroplasticity and recovery outcomes associated with these approaches. Therefore, it is crucial that we further investigate the efficacy of these and other methods to determine which rehabilitation approaches can offer maximal benefit for individuals recovering from stroke.

Recent advances in electroencephalography (EEG)-based brain-computer interface (BCI) offer new and potentially effective rehabilitative approaches to induce neural plasticity and restore motor function. These types of non-invasive BCI systems detect and translate a user’s electrophysiological signals into meaningful outputs in real-time to control external devices, such as computers or prosthetic devices. Importantly, these adaptive and personalized neurofeedback systems provide an alternative means of communication for patients with motor disabilities, as individuals can engage with the BCI system in a manner that is not contingent on peripheral motor control, effectively circumventing their impaired neuromuscular system. To date, many studies have observed clinical improvements in both upper limb motor function (Ang et al., 2015; Bajaj et al., 2015; Soekadar et al., 2015; Bundy et al., 2017; Ramos-Murguialday et al., 2019) and enhanced neural plasticity (Broetz et al., 2010; Mukaino et al., 2014; Ono et al., 2014) associated with BCI training. A number of BCI systems have been coupled with functional electrical stimulation (FES), a standard modality in stroke rehabilitation protocols. Electrical current is applied over paralyzed muscles to activate nerves and stimulate muscle contraction, with the goal to improve hand function and dexterity. Previous studies have shown that these integrated BCI-FES systems can foster recovery of both upper and lower limb function in the stroke survivor population (Daly et al., 2009; Do et al., 2012; Biasiucci et al., 2018; Cho et al., 2018; Tsuchimoto et al., 2019).

Resting-state functional magnetic resonance imaging (rs-fMRI) has gained widespread use as a powerful neuroimaging modality to probe and characterize brain connectivity with high spatial resolution. Resting-state functional connectivity (rsFC) measures temporal correlations between fluctuations in the spontaneous, low-frequency blood oxygenation level-dependent (BOLD) signal across distributed brain regions in a task-free setting. With rsFC, we can circumvent challenges with acquiring task fMRI data from patients with neurological diseases and study coactivating patterns that are consistent with and resemble functional networks active during tasks (Biswal et al., 1995). A large and growing number of studies have demonstrated the promise and utility of rsFC to characterize and monitor neural reorganization (Baker et al., 2014; Urbin et al., 2014), and yielded important clinical insights into the underlying pathophysiological mechanisms and effects of disease, as well as treatment response (Du et al., 2018). With its demonstrated use to study intrinsic brain connectivity dynamics, rsFC can serve as a means to monitor and evaluate the effects of stroke rehabilitation strategies on functional motor recovery.

Recently, we have shown that task-based functional connectivity and diffusion tensor imaging are useful in studying neural reorganization in patients with stroke who received BCI neurorehabilitation (Young et al., 2014b; Song et al., 2015). Several other works have reported beneficial effects in electrophysiological changes and functional motor recovery (Pichiorri et al., 2015; Bundy et al., 2017; Biasiucci et al., 2018; Remsik et al., 2019) associated with the use of BCI-controlled systems. Furthermore, Tsuchimoto et al. (2019) demonstrated motor network reorganization associated with the use of a BCI-FES system for restoring upper extremity motor function, however, their analysis was limited to functional connectivity between the somatosensory and motor cortices in the ipsilesional hemisphere. Other studies have investigated larger scale connectivity changes, in both spontaneous recovery and training-mediated stroke rehabilitation and observed increased activation in the contralesional and ipsilesional hemispheres separately and restoration of interhemispheric balance (Dodd et al., 2017). However, these underlying neuroplastic changes in interhemispheric and intrahemispheric rsFC in patients with stroke who undergo BCI intervention with FES are not fully understood. Even further, our understanding of how changes in rsFC relate to behavioral outcomes of motor ability with this form of BCI intervention is limited. Given that coordinated interactions among groups of regions underpin brain function and the underlying mechanisms of recovery processes, it is important to go beyond individual connections and investigate how rsFC network patterns relate to observed behavioral changes. In the stroke survivor population, there is considerable heterogeneity in stroke severity and degree of motor impairment, which invariably affect recovery potential. Therefore, it is critical that we have a detailed understanding of brain-behavior relationships associated with this intervention to both evaluate its therapeutic utility and further optimize neuromodulatory training to facilitate maximal motor recovery for patients after stroke.

### Overview of This Study

This aim of this study was to assess changes in rsFC and motor outcomes in patients of stroke with upper extremity motor deficits who completed EEG-based BCI intervention paired with FES. In the BCI paradigm, participants modulated sensorimotor rhythms, Mu (8–12 Hz) and Beta (18–25 Hz), using attempted hand movement to play a computer game while receiving multimodal feedback. Here, we performed group-level analysis of (1) changes in rsFC between brain regions involved in planning, initiating and executing motor commands and (2) changes in behavioral outcome measures related to motor function after BCI intervention. Results were subsequently used to identify correlations between observable changes in rsFC and behavioral improvements. Given previous findings of increased interhemispheric connectivity in spontaneous recovery and after treatment that correlated with motor recovery (Varkuti et al., 2013; Urbin et al., 2014; Fan et al., 2015), it was hypothesized that there would be significant increases in interhemispheric rsFC and behavioral performance from baseline to post-intervention following training with the BCI system In a similar vein, we hypothesized that these changes in rsFC between time points would correlate with gains in behavioral outcomes and have observable effects that persist 1 month after intervention.

## Materials and Methods

### Participants

Participants were recruited as part of an ongoing stroke rehabilitation study that is investigating the effects of EEG-based BCI with FES intervention on upper extremity motor recovery. The study was approved by Health Sciences Institutional Review Board of the University of Wisconsin-Madison and is registered with ClinicalTrials.gov with the assigned identifier, NCT02098265. Eligibility criteria were: (1) at least 18 years or older; (2) persistent upper extremity motor impairment resulting from ischemic or hemorrhagic stroke; (3) ability to provide written informed consent. Exclusion criteria were: (1) concomitant neurodegenerative or other neurological disorders; (2) psychiatric disorders or cognitive deficits that would preclude a subject’s ability to provide informed consent; (3) pregnant or likely to become pregnant during the study; (4) allergies to electrode gel, metal and/or surgical tape; (5) contraindications to MRI; (6) concurrent treatment for infectious disease. There was no cut-off requirement related to upper extremity motor impairment to participate in the BCI intervention. All subjects provided written informed consent prior to enrollment in the study.

In this study, the subject cohort was limited to patients who were in the chronic stage (>4 months since stroke onset), completed at least 9 of the 15 BCI intervention sessions, completed all 4 MRI scans and neuropsychological assessments and had neuroimaging data obtained from 3T MRI scanners. Furthermore, subjects were excluded here if they presented with bilateral lesions, as additional variables could be introduced that confound the analysis. In total, 23 participants (age = 62 ± 12.8 years, 10 females) who completed BCI intervention were included in the current analysis. The average time since stroke, defined as the duration between date of stroke onset and the preliminary visit, for subjects in the cohort was 33 ± 40.5 months. Severity of upper extremity motor impairment was evaluated based on performance on the Action Research Arm Test (ARAT) (Carroll, 1965) at the preliminary visit and classified as follows: mild = 40–57 (*n* = 7), moderate = 20–40 (*n* = 2), severe = 0–20 (*n* = 14). Post-stroke handedness was assessed using the Edinburgh Handedness Inventory (Oldfield, 1971). 19 subjects were right-handed, 2 were left-handed and 2 were ambidextrous. Demographic and clinical information about the participants are summarized in Table 1.

**TABLE 1:** Participant demographics.

**Subject**	**Age range (years)**	**Gender**	**Lesion side**	**Lesion location**	**Time since stroke (months)**
1	50–54	M	L	MCA	15
2	59–63	F	L	Frontal lobe	6
3	64–68	M	L	MCA	24
4	71–75	F	L	MCA	16
5	57–61	M	L	MCA	28
6	43–47	F	R	MCA	99
7	69–73	F	R	MCA	26
8	78–82	M	R	Occipital lobe	21
9	74–78	M	R	MCA	168
10	41–45	M	L	MCA	6
11	62–66	F	R	Frontal lobe	13
12	69–73	M	R	MCA	26
13	73–77	F	R	Putamen	23
14	46–50	M	R	Pons	4
15	54–60	M	L	MCA	12
16	48–52	M	R	MCA	16
17	75–79	M	L	PVWM	22
18	67–71	M	R	Putamen	90
19	81–85	F	L	Cerebellar vermis	19
20	72–76	F	R	Prefrontal	6
21	40–44	F	R	Frontal parietal	87
22	55–59	F	R	Frontal lobe	19
23	45–49	M	R	ATL	15

*F, female; M, male; L, left; R, right; MCA, middle cerebral artery; PVWM, periventricular white matter; ATL, anterior temporal lobe; ARAT, Action Research Arm Test; Time since stroke was calculated as ([Date of Stroke − Study Enrollment Date]/365 days) × 12 months.*

### Study Design

**FIGURE 1:**
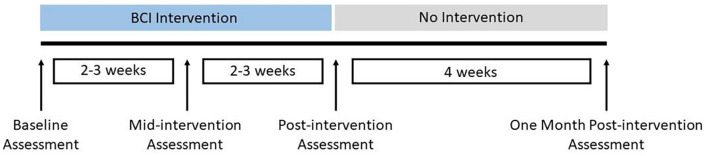
Study schedule for BCI intervention.

This ongoing study has employed a permuted block randomization scheme of which details have been described previously (Mohanty et al., 2018). In the present work, we only report results based on analysis of neuroimaging and behavioral data from all subjects during from the intervention phase, which includes three distinct time points, pre-intervention, post-intervention and 1 month post-intervention. The study schedule for the BCI intervention is shown in Figure 1. This is line with the main focus of the current study, which was based on a within-subjects design, where each subject serves as his/her own control based on baseline scores, to monitor changes in functional connectivity and behavioral outcomes over time associated with BCI intervention.

### Brain-Computer Interface Intervention

**FIGURE 2:**
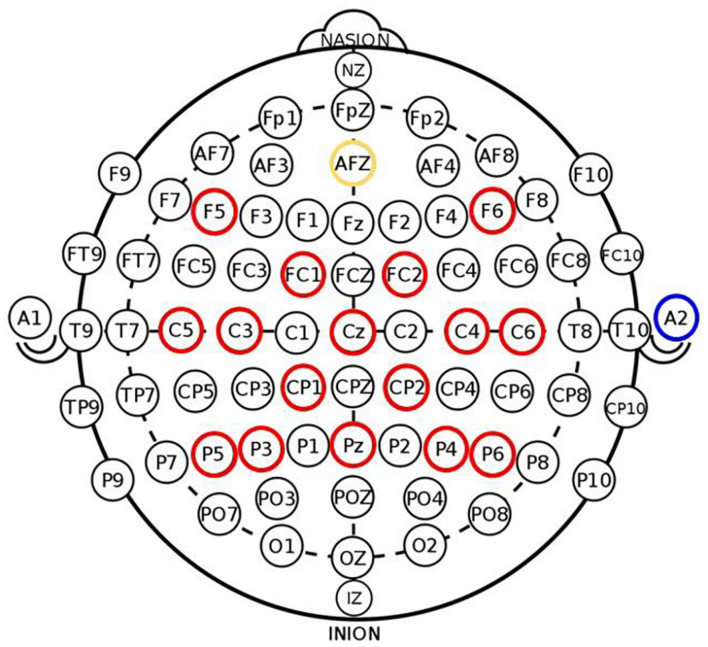
Electrode placement in 16-channel EEG cap used in BCI system (red—active electrodes, yellow—ground electrode, blue—reference site on right ear lobe) adapted from Remsik et al. (2019). Signals from electrode channels C3 and C4 served as input into the BCI classifier to control lateral cursor movement.

All subjects received up to 15 2-h EEG-based BCI sessions with visual feedback and FES that occurred 2–3 times per week. The BCI system and intervention procedure are consistent with those detailed in previous studies (Wilson et al., 2009; Young et al., 2014a). Briefly, BCI interventions were administered on a computer using BCI2000 software (Schalk et al., 2004) version 2. Modifications were made to the system to incorporate tongue stimulation (Tongue Display Unit 0.130 Wicab Inc.) and FES using an LG-7500 Digital Muscle Stimulator (LGMedSupply, Cherry Hill, NJ, United States; Arduino 1.0.4). However, due to equipment-related issues, very few subjects in the overall larger study received tongue stimulation, therefore the analysis and results reported here only pertain to BCI intervention with FES. The present subject cohort includes two individuals who received some tongue stimulation during one or two intervention sessions, however, it is noted their data were not outliers with respect to neuroimaging and behavioral measures in a manner that could skew results and were thus included in the analysis to improve overall statistical power. EEG data were acquired using a g.GAMMA cap and amplifier (Guger Technologies), with 16 active electrodes (F5, FC1, C5, C3, CP1, P5, P3, Cz, Pz, F6, FC2, C4, C6, CP2, P4, and P6) and a reference site at the right ear lobe as shown in Figure 2. The system was configured according to the standard 10–20 system of electrode placement. Within BCI2000, raw EEG signals were preprocessed using a band-pass filter (0.1–100 Hz) and a notch filter to remove noise. The power spectrum was estimated by fitting an autoregressive model, and extracted features in Mu (8–12 Hz) and Beta (18–25 Hz) from electrodes C3 and C4 during cued voluntary movement of the left and right hand were used as input into a linear classifier to determine lateral cursor movement.

BCI intervention sessions consisted of three parts: (1) an open-loop calibration task without any feedback, (2) a closed-loop task with visual feedback, and (3) a closed-loop task with visual feedback, tongue stimulation and FES. During the open-loop calibration task, subjects were prompted with visual and auditory cues to execute right/left hand movement, imagine right/left hand movement or rest. Given that motor deficits related to grasping and releasing objects are common in patients of stroke, subjects chose between either multi-finger extension or flexion hand movement for the intervention. Calibration was performed at the beginning of each session to account for slight variability in Mu and Beta rhythms across individuals. During the calibration task, EEG activity were recorded from subjects as they performed attempted left and right hand movement to identify activation patterns in the sensorimotor cortex corresponding to voluntary movement of each hand. These patterns were saved as the EEG-based control signals for the following closed-loop task. Subjects were instructed to perform attempted movement during both the calibration and closed-loop tasks to simulate real-world tasks that they would engage in on a daily basis.

Following the calibration task, subjects performed the closed-loop task, which consisted of a cursor task game. The goal was to move a cursor (ball) toward a rectangular target that was randomly positioned on either the left or right side of the computer screen in each trial. Subjects were instructed to perform either multi-finger extension or flexion of their right or left hand to elicit real-time EEG control signals identified during calibration to control lateral cursor movement (left or right) toward the target. Here, cursor movement served as continuous visual feedback to the subject. The closed-loop task with visual feedback consisted of a minimum of 10 successful runs (8–12 trials per run), which subjects had to complete with at least 70% accuracy. If performance accuracy was less than 70% after these runs, individuals completed additional trials until they consistently reached or exceeded the necessary level of proficiency before transitioning to the next task. In the present analysis, all subjects achieved the required 70% accuracy within the first 10 runs of the closed-loop task.

Following the runs with visual feedback alone, sensorimotor rhythm-triggered FES was introduced into the closed-loop cursor task. The coupling of FES with BCI creates a direct communication pathway between the brain and peripheral stimulation device, effectively “closing the loop” between the brain and impaired muscles. FES was delivered through two 2” × 2” square electrodes that were placed on either the flexor digitorum superficialis of the subject’s forearm stimulate multi-finger flexion or the extensor digitorum communis to stimulate finger extension. The FES pulse frequency was set to 60 Hz to generate tetanic contraction with a pulse width = 150 μs and could be adjusted in increments of 0.5 mA based on the subject’s comfort level. During trials in which the target appeared on the side of the affected arm, if EEG signals corresponding to multi-finger extension or flexion of the affected arm were detected, FES was administered to the subject. Thus, in this construction, the BCI system links the modulation of brain activity to concurrent sensory feedback. Game settings, such as target size and cursor speed, could be adjusted to vary task difficulty in order to keep subjects engaged and motivated throughout the session.

### Data Acquisition: Neuroimaging

MRI scans were acquired on GE 750 3T MRI scanners (GE Healthcare, Waukesha, WI) with an 8-channel head coil. 5-min T1-weighted anatomical scans were obtained using a BRAVO FSPGR sequence: TR = 8.16 ms, TE = 3.18 ms, TI = 450 ms, FOV = 256 mm, matrix size = 256 × 256, flip angle = 12°, number of slices = 156, and slice thickness = 1 mm. For rs-fMRI scans, subjects were instructed to remain relax and awake with their eyes closed. 10-min rs-fMRI data were acquired using a T2^∗^-weighted gradient—echo planar imaging (EPI) pulse sequence: 231 volumes, TR = 2,600 ms, TE = 22 ms, FOV = 224 mm, matrix size = 64 × 64, flip angle = 60°, 40 axial slices, and 3 × 3 × 3 mm^3^ voxels.

### Data Acquisition: Behavioral Outcome Measures

To assess the behavioral effects of BCI intervention, a neuropsychological battery of objective and subjective measures was administered to each participant at each time point. The primary outcome measures were the ARAT (Carroll, 1965; Lang et al., 2006) and 9-Hole Peg Test (9-HPT) (Chen et al., 2009). Scores on the ARAT, a widely used 19-measure metric quantifying upper extremity motor function in stroke recovery, were reported as total points scored out of 57 when the participant performed the task with his/her affected arm. In ARAT, the minimal detectable change (MDC) and minimally clinically important difference (MCID) were 3 points and 5.7 points, respectively (Van der Lee et al., 1999). 9-HPT is a quantitative assessment that measures finger dexterity, and scores were calculated as the average of two timed trials using the affected arm. Secondary outcome measures included the Stroke Impact Scale (SIS) (Duncan et al., 1999; Carod-Artal et al., 2008) standard domains, Strength, Activities of Daily Living (ADL), Mobility and Hand Function (HF), and Barthel Index (Mahoney and Barthel, 1965). Following standard SIS scoring practices, SIS domain scores were scaled to adjust for the lowest possible individual raw score and raw score range.

### Data Preprocessing

Neuroimaging data were preprocessed using AFNI (Cox, 1996) and FSL (FMRIB Software Library^1^. Preprocessing steps included removal of the first three volumes of each scan, image despiking, slice time correction, alignment with anatomical scan, spatial smoothing at 4 mm with a full width at half maximum Gaussian kernel, transformation into MNI space (3.5 mm isotropic), motion censoring (per TR motion > 1 mm or 1°), nuisance regression (regressing out the signal from white matter) and bandpass filtering (0.009–0.08 Hz). Given the ongoing controversy of global signal regression, it was not included as a preprocessing step in this work. To account for heterogeneity in lesion location among subjects, MRI scans of a left hemisphere stroke and motor impairment on the contralateral side were mirrored along the midline to generate scans of a right hemisphere stroke lesion. Thus, as a cohort, the stroke lesion was modeled in the right hemisphere, and the motor impairment was in the left upper extremity. This additional preprocessing step of mirroring MRI scans was based on the inherent assumption of symmetry in motor network activity and organization and as such are comparable as performed in previous studies (Ward et al., 2003; Stagg et al., 2012; Young et al., 2014).

### Functional Connectivity Analysis

Motor network regions of interest (ROIs) analyzed in this work are cortical and subcortical regions that are activated during visually paced hand movements and are based on previous studies that investigated rsFC changes in participants with stroke (Grefkes et al., 2008; Nair et al., 2015b). The eight ROIs were: left primary motor cortex (L. M1) (MNI coordinates: −39, −22, 57), right primary motor cortex (R. M1) [(MNI coordinates: 40, −23, 55), left premotor cortex (L. PMC) (MNI coordinates: −48, 1, 36), right premotor cortex (R. PMC) (MNI coordinates: 58, 1, 35), left supplementary motor area (L. SMA) (MNI coordinates: −6, −14, 53), right supplementary motor area (R. SMA) (MNI coordinates: 8, −14, 52), left thalamus (L. Thal) (MNI coordinates: −8, −26, 12), and right thalamus (R. Thal) (MNI coordinates: 8, −26, 12)]. Henceforth, ROIs located in the right hemisphere are denoted with the prefix “i” for the ipsilesional hemisphere, and ROIs located in the left hemisphere are denoted with the prefix “c” for the contralesional hemisphere. MNI coordinates for each ROI were used to create 8-mm radius spherical seeds and generate a mask for each motor network region. For each subject, fMRI BOLD timeseries data was extracted from the regions, and Pearson correlation was computed between all pairs of ROIs to compute correlation connectivity matrices. In total, there were [8 × (8−1)]/2 = 28 pairwise connections. Here, each *(i,j)* element of the correlation matrix represented the strength of association or connectivity between the *i*th and *j*th ROI. Fisher’s *r*-to-*z* transform was applied to the correlation matrices to stabilize variance in the data, generating subject-specific z-score matrices, which were used for group-level analysis using the Network-Based Statistic (NBS) (Zalesky et al., 2010) toolbox.

### Group-Level Analysis

With the aim to investigate the effects of BCI intervention on motor network functional connectivity at the group-level, we combined NBS with generalized estimating equations (GEE) (Hanley et al., 2003) to identify statistically significant connections at each time point and assess how they changed over time. We first briefly describe the procedure for performing group comparisons of functional connectivity using NBS, a non-parametric approach that identifies subnetworks of functionally connected ROIs. First, mass univariate *t-*tests are performed on each pairwise connection to compute a corresponding t-statistic. NBS then generates a sparse graph containing only connections that exceed a predefined *t*-statistic threshold, termed suprathreshold connections, and uses a breadth-first search to identify connected components within the subset identified. Permutation testing is performed on the components to identify subnetworks that are that are statistically significant such that we can reject the null hypothesis of a zero mean, and a family-wise error-corrected *p*-value is calculated for each subnetwork of connections deemed significant. Full details of this method can be found in Zalesky et al. (2010). Here, we used NBS with one-sample *t*-tests (*t*-statistic threshold = 2.0, *p* < 0.05, permutations = 5,000) run on the z-score matrices to identify statistically significant connections at the group-level for each of the three time points. For each subject, the mean z-score was calculated by averaging the strengths of the significant connections identified at each time point. Similar to previous work that investigated global changes in intrahemispheric and intrahemispheric functional connectivity after stroke (Nair et al., 2015a; Lee et al., 2018), connections were organized into intrahemispheric connectivity (connections within the same hemisphere), interhemispheric connectivity (connections between both hemispheres), and network connectivity (combined intrahemispheric and interhemispheric connections) for each subject and time point for subsequent analysis.

Additional group-level analyses of rsFC and behavioral outcome measures were performed using GEE with a significance threshold of *p* < 0.05. GEE, an extension of the generalized linear model, is a semi-parametric approach for longitudinal analysis of correlated continuous or categorical response variables, in which there are no underlying assumptions related to the distribution of the data (i.e., normal, binomial, etc.). Unlike the mixed effects model, which uses random effects to quantify correlation between repeated measures at the individual-level, GEE uses changes in the mean group responses to generate population averaged models. “Time since stroke” (months) and baseline scores for each assessment were included as covariates. Changes in motor network rsFC were further analyzed to identify correlations with changes in behavioral outcomes from pre-intervention to both post- and 1-month post-intervention. Subjects that had a change in rsFC ≥ two standard deviations away from the mean and/or exhibited ceiling or floor effects were deemed outliers and excluded in the group-level analyses.

## Results

### Functional Connectivity in the Motor Network

At baseline, one-sample *t*-tests revealed that the top three significant connections identified by NBS at the group level were: i. M1 – i. SMA (*t*-statistic = 8.12), i. M1 – c. SMA (*t*-statistic = 8.14), and c. SMA – i. SMA (*t*-statistic = 10.48). At post-intervention, the three strongest connections included: i. M1 – c. SMA (*t*-statistic = 9.06), i. M1 – i. SMA (*t*-statistic = 9.17), and c. SMA – i. SMA (*t*-statistic = 10.30). At 1-month post-intervention, the top three significant connections identified were: i. M1 – c. SMA (*t*-statistic = 7.98), i. M1 – i. SMA (*t*-statistic = 8.45), and c. SMA – i. SMA (*t*-statistic = 10.77). NBS identified 15 significant connections at each time point, which are grouped at the interhemispheric and intrahemispheric level in Table 2. To examine changes in rsFC in the motor network changed after intervention, we investigated how the significance of connections changed from baseline to post- and 1-month post-intervention.

**TABLE 2:** Significant interhemispheric and intrahemispheric connections at each time point identified using NBS (Zalesky et al., 2010) based on *t*-statistic (*p* < 0.05).

	**Pre- *p*-value = 0.0034**	**Post- *p*-value = 0.0064**	**One month Post- *p*-value = 0.007**
**Interhemispheric connections**			
c. PMC – i. SMA	2.98	4.25	3.96
c. M1 – i. PMC	3.24	4.65	4.27
i. M1 – c. PMC	3.55	4.66	3.07
c. PMC – i. PMC	4.02	5.07	4.03
c. M1 – i. SMA	4.99	5.57	5.65
i. PMC – c. SMA	5.20	6.50	5.77
c. M1 – i. M1	5.30	6.15	6.06
i. M1 – c. SMA	8.14	9.06	7.98
c. SMA – i. SMA	10.48	10.30	10.77
**Intrahemispheric connections**			
c. M1 – c. PMC	3.08	3.75	4.47
c. PMC – c. SMA	3.38	4.26	4.14
i. PMC – i. SMA	5.57	6.47	5.69
c. M1 – c. SMA	6.89	7.16	7.17
i. M1 – i. PMC	7.14	7.53	7.32
i. M1 – i. SMA	8.12	9.17	8.45

*Pre-, Pre-intervention; Post-, Post-intervention; One-month post-, One-month post-Intervention; i., ipsilesional; c., contralesional.*

As some connections increased in strength while others decreased throughout and after intervention, it is not surprising that paired *t*-tests of connections using permutation testing from baseline to each time point did not reveal significance. However, GEE analysis revealed significant group-level increases in rsFC from baseline to immediately post-intervention for average network connectivity (*p* = 0.000000392), intrahemispheric connectivity (*p* = 0.01), and interhemispheric connectivity (*p* = 0.026). Furthermore, there was a markedly significant increase in average network connectivity strength that persisted from pre- to 1 month-post-intervention (*p* = 0.000358), but not for intrahemispheric connectivity (*p* = 0.053) or interhemispheric connectivity (*p* = 0.198).

### Behavioral Outcomes Analysis

Group-level analysis of behavioral measures using GEE revealed significant improvements on SIS ADL (*p* = 0.044) and SIS Mobility (*p* = 0.041) from baseline to post-intervention. Furthermore, there was a trend toward significance for improvement in Barthel Index (*p* = 0.057) from baseline to post-intervention. While 3 subjects improved by MDC (3 points) or MCID (5.7 points) on ARAT from pre-intervention to post-intervention, there was not a significant increase on ARAT as a group. However, from baseline to 1 month post-intervention, participants significantly improved on ARAT using the affected arm (*p* = 0.023), with 6 subjects improving by MDC or MCID. In addition, patients exhibited significant increases in SIS Strength (*p* = 0.013) and SIS Mobility (*p* = 0.019) and a trend toward significance on SIS ADL (*p* = 0.062) between time points. It should be noted that group performance on ARAT was only analyzed from subjects that could perform the assessment with the affected arm (*n* = 19). Full results of group-level changes and improvement scores in primary and secondary outcome measures from pre-intervention to post- and 1 month post-intervention are presented in Table 3.

**TABLE 3:** GEE analysis of behavioral outcome measures.

**Outcome measure**	**Time**	**N**	**ImprovementMean (SD)**	**GEE *p*-value**
Barthel index	Pre- to post- Pre- to 1 month post-	23 23	2.3 (2.2) 5.7 (7.8)	0.057* 0.180
SIS strength	Pre- to post- Pre- to 1 month post-	23 23	4.9 (14.2) 7.1 (13.7)	0.097* **0.013**
SIS ADL	Pre- to post- Pre- to 1 month post-	23 23	4.6 (10.9) 4.3 (11.2)	**0.044** 0.062*
SIS mobility	Pre- to post- Pre- to 1 month post-	23 23	5.2 (12.2) 6.1 (12.5)	**0.041 0.019**
SIS hand function	Pre- to post- Pre- to 1 month post-	23 23	2.0 (11.5) 2.5 (11.6)	0.414 0.303
ARAT (affected)	Pre- to post- Pre- to 1 month post-	19 19	1.6 (5.2) 3.0 (5.6)	0.187 **0.012**
9-HPT (affected)	Pre- to post- Pre- to 1 month post-	7 7	−0.16 (16.1) −2.6 (8.7)	0.413 0.431

*0.05 < p < 0.1.

*Pre-, Pre-intervention; Post-, Post-intervention; One-month post-, One-month post-intervention; SIS, Stroke Impact Scale; ADL, Activities of Daily Living; ARAT, Action Research Arm Test; 9-HPT, 9-Hole Peg Test.*

*Bold values indicate *p*-values < 0.05.*

### Associations Between Changes in Functional Connectivity and Behavioral Outcome Measures

**FIGURE 3:**
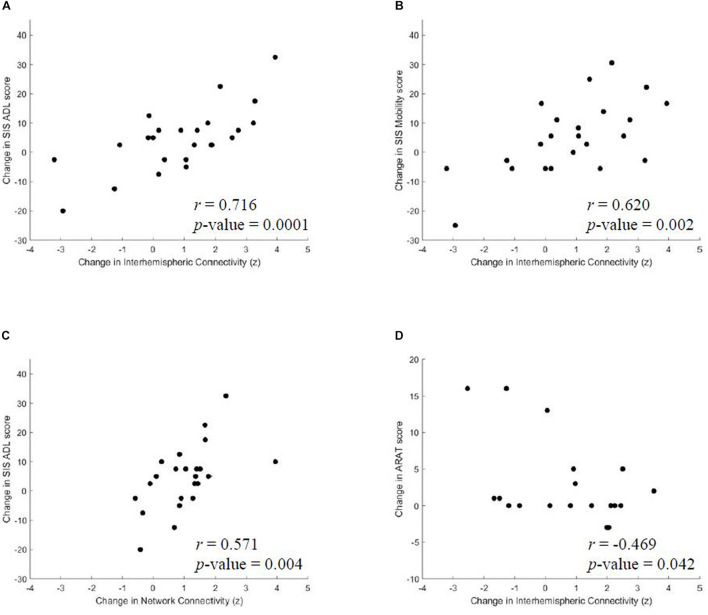
From pre-intervention to post-intervention, significant correlations were identified between changes in **(A)** SIS ADL and interhemispheric connectivity, **(B)** SIS Mobility and interhemispheric connectivity and **(C)** SIS ADL and average network connectivity. **(D)** From pre-intervention to 1 month post-intervention, changes in ARAT negatively correlated with interhemispheric connectivity changes. SIS, Stroke Impact Scale; ADL, Activities of Daily Living.

Previous studies primarily focused on changes in interhemispheric functional connectivity following stroke rehabilitation (Varkuti et al., 2013; Young et al., 2014; Fan et al., 2015), however, we explored network, intrahemispheric and interhemispheric dynamics for subsequent group-level correlation analysis with behavioral performance. From baseline, or pre-intervention, to post-intervention, we identified positive correlations between improvements in SIS ADL and increases in average network connectivity (*r* = 0.571, *p* = 0.004) and functional gains in SIS ADL (*r* = 0.716, *p* = 0.0001) and SIS Mobility (*r* = 0.620, *p* = 0.002) with changes in interhemispheric rsFC, which are shown in Figure 3. Moreover, there was a trend toward significance in correlation between interhemispheric connectivity and 9-HPT for the affected arm (*r* = −0.695, *p* = 0.083) to post-intervention. No correlations were identified between intrahemispheric connectivity and outcome measures from baseline to any time point. From baseline to 1 month post-intervention, changes in interhemispheric connectivity negatively correlated with ARAT (*r* = −0.469, *p* = 0.042). Results of correlation analyses of rsFC and behavioral changes after BCI intervention are listed in Table 4.

**TABLE 4:** Correlations between change in average and interhemispheric rsFC and behavior.

	**N**	**Pearson’s *r***	***p*-value**
**Average network connectivity Pre-intervention to post-intervention**			
SIS strength	23	0.329	0.125
SIS ADL	23	0.571	**0.004**
SIS mobility	23	0.243	0.263
SIS hand function	23	–0.021	0.924
ARAT (affected)	19	0.226	0.352
9-HPT (affected)	7	–0.508	0.224
**Average network connectivity Pre-intervention to 1 month post-intervention**			
SIS strength	23	0.150	0.494
SIS ADL	23	–0.268	0.217
SIS mobility	23	0.176	0.442
SIS HF	23	–0.352	0.100
ARAT (affected)	19	–0.273	0.258
9-HPT (affected)	7	–0.259	0.575
**Interhemispheric connectivity Pre-intervention to post-intervention**			
SIS strength	23	0.206	0.346
SIS ADL	23	0.716	**0.0001**
SIS mobility	23	0.620	**0.002**
SIS hand function	23	0.301	0.163
ARAT (affected)	19	0.034	0.891
9-HPT (affected)	7	–0.695	0.083*
**Interhemispheric Connectivity Pre-intervention to 1 month post-intervention**			
SIS strength	23	0.072	0.743
SIS ADL	23	0.01	0.965
SIS MOBILITY	23	0.084	0.703
SIS hand function	23	–0.257	0.237
ARAT (affected)	19	–0.469	**0.042**
9-HPT (affected)	7	–0.398	0.377

*0.05 < p < 0.1. Bold values indicate *p*-values < 0.05.

## Discussion

The objective of this study was to evaluate the effects of EEG-based BCI intervention with FES on intrinsic connectivity dynamics and upper limb motor recovery post-stroke. We showed that from baseline to post-intervention, there were significant changes in network, intrahemispheric and interhemispheric connectivity and improvements in objective and patient-reported measures that persisted to 1 month post-intervention. Notably, from baseline to post-intervention, changes in interhemispheric connectivity correlated with gains in SIS ADL and Mobility. Furthermore, interhemispheric connectivity changes negatively correlated with ARAT from baseline to 1 month after intervention.

### Functional Connectivity Changes Associated With Intervention

We see BCI-training associated changes in rsFC at the connection level from baseline to post- and 1-month post-intervention, as indicated by strengthening and weakening of connections based on increases and decreases in their *t*-statistics, respectively. In particular, the contralesional and ipsilesional supplementary motor areas and primary motor cortex showed significantly increased interhemispheric and intrahemispheric (within the ipsilesional hemisphere) coupling of BOLD activity, which aligns with previous evidence of improvement in upper-limb recovery following BCI intervention (Varkuti et al., 2013). In particular, the ipsilesional primary motor cortex was identified in several significant connections across time points, which is consistent with published studies that found it to be a main target for stroke neurorehabilitation (Buetefisch, 2015; Tsuchimoto et al., 2019). Moreover, the supplementary motor areas have been shown to play a functional role in both motor imagery and motor planning (Min et al., 2020), therefore, increases in rsFC strength exhibited between the ipsilesional and contralesional supplementary motor areas after BCI training may be a form of adaptive motor network reorganization. In addition, connectivity between the ipsilesional and contralesional primary motor cortex strengthened after intervention, which supports previous findings of increases in M1-M1 connectivity after rehabilitation that correlated with improved recovery outcomes (Fan et al., 2015; Min et al., 2020). Furthermore, connectivity analysis revealed asymmetry in rsFC, with more significant connections identified in the contralesional hemisphere than in the ipsilesional hemisphere. This may indicate a greater role of the contralesional hemisphere in neural plasticity related to motor recovery, given the severity and extent of stroke-induced damage in the ipsilesional hemisphere.

As hypothesized, there were notable increases in network and interhemispheric functional connectivity from baseline to post-intervention. These results are consistent with recent findings demonstrating associations between decreased interhemispheric rsFC and motor impairment that significantly increase during post-stroke motor recovery (Park et al., 2011; Fan et al., 2015). Therefore, BCI intervention may be beneficial in strengthening functional connections to restore sensorimotor control. Currently, there is no definitive consensus on how to optimally activate or inhibit the contralesional or ipsilesional hemisphere for stroke recovery (Dodd et al., 2017), however, these findings provide evidence to explore targeted interventions involving interhemispheric connectivity to foster neuroplasticity for regaining motor function. Nevertheless, future studies that assess rsFC interhemispheric and intrahemispheric dynamics throughout intervention will more comprehensively elucidate the roles of the contralesional and ipsilesional hemispheres in stroke recovery.

### Improvements in Behavioral Outcomes Associated With Intervention

Our results also showed significant group-level improvements in outcome measures, including ARAT and SIS domains, that are preserved long-term after completing BCI intervention. Notably, behavioral improvements in ARAT of MDC/MCID was observed in three subjects from baseline to post-intervention and 6 subjects from baseline to 1 month post-intervention. This suggests that BCI-mediated intervention may have therapeutic benefits for individuals with varying degrees of motor deficits that are quantifiable by standardized objective measures. It is acknowledged that some individuals were unable to perform the primary outcome measures due to severity of motor impairment or were excluded due to floor/ceiling effects, which affected statistical power in group-level analyses. Furthermore, the observed improvement in SIS domains is promising, as this may indicate that subjects believe that they are regaining autonomy in daily activities and have improved quality of life after participating in BCI interventions. These results should be further validated using additional objective metrics, such as the Fugl-Meyer Assessment, that can more holistically evaluate motor impairment and recovery after intervention.

### Relationship Between Changes in Functional Connectivity and Functional Outcomes

We then aimed to identify brain-behavior correlations based on functional interactions in the motor network responsible for planning and execution of hand movement at the network and interhemispheric level. Notably, increases interhemispheric connectivity positively correlated with gains in SIS ADL and Mobility from baseline to post-intervention, which is line with previous work that demonstrated the predictive value of interhemispheric rsFC for upper limb motor recovery after stroke (Min et al., 2020). Furthermore, disruptions in coordinated interhemispheric connectivity has been shown to be associated with impaired upper extremity motor function after stroke (Murase et al., 2004; Carter et al., 2010; Dimyan and Cohen, 2011). Hence, the observed increases in rsFC may be indicative of neural reorganization supporting post-stroke motor recovery. In addition, changes in interhemispheric connectivity negatively correlated with improvements in ARAT from baseline to 1 month post-intervention. This may suggest that sustained effects of intervention are evident in behavioral improvements, however, cortical reorganization is occurring again after discontinuation of BCI intervention. Future studies should focus on disentangling this to determine if more frequent or regular participation in BCI intervention are required to induce sustained changes in both neuroplasticity and motor function. Interestingly, several severely impaired patients were unable to perform the ARAT or 9-HPT, however, they exhibited higher increases in interhemispheric and average network connectivity that correlated with larger improvements in SIS domains relative to mild and moderate patients. It is possible that the present objective measures may not be sufficient for assessing motor function across all degrees of motor deficits, albeit these findings provide evidence that patients with severe upper extremity impairments can receive some beneficial effects from BCI intervention based on the measures evaluated here. In addition, the trend toward significance in correlation between interhemispheric rsFC and 9-HPT from baseline to post-intervention may indicate that BCI training is beneficial for improving hand dexterity. However, the current analysis was limited in statistical power due to the number of subjects able to complete the task. It is worth noting that patients included in the analysis here were ≥ 4 months post-stroke, therefore, it is unlikely that spontaneous neurobiological recovery confounded any observed changes associated with the intervention. Overall, these results suggest that linking BCI training with somatosensory feedback may be an effective restorative therapy that can promote neuroplasticity and functional upper limb motor recovery after stroke.

It is important to emphasize that patient-reported measures are valuable in evaluating the impact of stroke and treatment, as they can overcome the limitations of floor or ceiling effects commonly observed with standard scales, such as ARAT, Barthel Index, and Fugl-Meyer Assessment. Moreover, these subjective measures can be sensitive to quantifying the extent and impact of stroke and rehabilitation in patients with minimal or severe impairment that may otherwise not be measurable with standard scales (Stewart and Cramer, 2013). Furthermore, patient-reported measures provide important insights into disease effects across domains of health that impact patients’ daily activities and afford a more comprehensive understanding of patient perception of functional status and recovery progress (Richardson et al., 2016; Katzan et al., 2017). Overall, the correlations between increased motor network connectivity and outcome measures suggest that functional reorganization associated with BCI intervention may reflect improvements in patient ability to participate in daily motor-related tasks and enhanced quality of life. It is possible that in the current population analyzed, these effects cannot be fully captured using clinical measures that require fine motor control. Nonetheless, these findings suggest that interhemispheric interactions within the motor network correlate with behavioral improvements and should be targeted for future optimization of BCI training with FES to facilitate neurological and upper extremity motor recovery for patients after stroke.

### Limitations

This study has a number of limitations that should be noted. The analysis was based on a relatively small sample size with a heterogeneous patient population in terms of time since stroke, stroke lesion location and degree of upper extremity impairment. In addition, several subjects had severe motor impairments that precluded them from completing the objective assessments, which reduced statistical power in group-level analyses. However, it is important to note that challenges related to patient recruitment and retention for these longitudinal studies invariably limits the sample size that can be assessed to discern therapeutic effects on stroke recovery. Even so, the size of the patient cohort analyzed here is considerably larger than similar studies in the literature. Nonetheless, these findings were robust enough to show significant changes in rsFC and behavioral outcome measures after BCI intervention, with a number of subjects exhibiting meaningful clinical improvements in functional outcomes. This may suggest that subjects with varying degrees of motor impairment can likely benefit from this form of BCI rehabilitation to regain autonomy in daily life. Future studies should focus on a larger and more homogeneous population to both replicate and validate the present results and delineate the effects of BCI intervention on different subgroups within the stroke survivor population. This could inform who can optimally benefit from BCI intervention and predict recovery potential based on chronicity and/or severity of motor deficits. Another limitation to consider is that functional connectivity was investigated in eight cortical regions involved in motor planning and execution. Other motor-related regions, such as the sensorimotor cortex, middle temporal gyrus, middle frontal gyrus, putamen, and caudate, are likely involved in neuroplasticity changes underlying motor recovery and should be included in future analyses. This would provide a deeper understanding of the underlying neurophysiological mechanisms of rsFC changes that could elucidate both adaptive and maladaptive brain and behavioral changes related to BCI training. Furthermore, because the focus of this current analysis was to track changes in functional connectivity and behavior over time within subjects, no control group was included. However, this could be done in a future study when a sufficient number of control subjects have completed the BCI intervention protocol. In addition, it is acknowledged that factors, such as motivation to participate in research, practice effects, or repetitive use of the paretic arm in a supervised setting may have led to some of the observed changes, rather than the neurofeedback in the BCI intervention. Nonetheless, this work provides evidence that it is possible to improve motor-related outcomes in patients with chronic phase stroke, and BCI intervention may be beneficial in promoting that recovery. Taken together, the results presented here provide new evidence that it is possible to promote neuroplasticity changes and improve motor-related outcomes in patients of stroke in the chronic phase, and BCI intervention with FES may be beneficial in facilitating functional motor recovery after stroke.

## Conclusion

The current study provides new evidence that suggest that non-invasive EEG-based BCI with FES intervention can facilitate changes in interhemispheric interactions and improve behavioral outcomes for patients of stroke with upper extremity impairment. The present findings are important as they indicate that patients may have functional capacity to restore motor function in the chronic stage of stroke that can be fostered through BCI intervention with somatosensory feedback, which could improve overall autonomy in daily life for survivors. Findings also build on previous results and demonstrate a relationship between changes in interhemispheric rsFC and motor improvements when evaluating BCI-mediated effects on motor recovery after stroke. Overall, the results presented here open the door to future avenues of research and customized optimization of the neuromodulatory training to facilitate cortical reorganization and improve motor recovery outcomes in patients after stroke.

## Data Availability Statement

The raw data supporting the conclusions of this article will be made available by the authors, without undue reservation.

## Ethics Statement

The studies involving human participants were reviewed and approved by the Health Sciences Institutional Review Board of the University of Wisconsin-Madison. The patients/participants provided their written informed consent to participate in this study.

## Author Contributions

AS designed the experiments, performed the analysis, and drafted the manuscript. VN was involved in designing the experiments, interpreting the results, and drafting the manuscript. VP was a PI on the study and was involved in study design, intellectual content, and supervising the entire study. All authors approved the final version of the submitted manuscript.

## Conflict of Interest

The authors declare that the research was conducted in the absence of any commercial or financial relationships that could be construed as a potential conflict of interest.

## Publisher’s Note

All claims expressed in this article are solely those of the authors and do not necessarily represent those of their affiliated organizations, or those of the publisher, the editors and the reviewers. Any product that may be evaluated in this article, or claim that may be made by its manufacturer, is not guaranteed or endorsed by the publisher.

## Footnotes

1
www.fmrib.ox.ac.uk/fsl
